# Familial multiple lipomas coexisting with celiac disease: a case report

**DOI:** 10.1186/1752-1947-8-309

**Published:** 2014-09-16

**Authors:** Ilyas Sayar, Levent Demirtas, Mehmet Gurbuzel, Arda Isik, Kemal Peker, Baris Gulhan

**Affiliations:** 1 Pathology Department, Erzincan University, Erzincan, Turkey; 2 Internal Medicine Department, Erzincan University, Erzincan, Turkey; 3 Molecular Biology Department, Erzincan University, Erzincan, Turkey; 4 General Surgery Department, Erzincan University, Erzincan, Turkey; 5 Microbiology Department, Erzincan University, Erzincan, Turkey

**Keywords:** Celiac disease, Familial multiple lipomas

## Abstract

**Introduction:**

Gluten enteropathy (celiac disease) is a chronic disease and presents as diarrhea, weight loss and anemia.

**Case presentation:**

A 35-year-old Caucasian man with gluten enteropathy, familial multiple lipomas and seborrheic keratosis was seen in our clinic. After confirmation of the diagnosis, he was advised to follow a gluten-free diet. His clinical improvement was evaluated and confirmed with biopsy.

**Conclusion:**

Celiac disease is known to be associated with many systemic diseases and skin lesions but its association with familial multiple lipomas has not yet been reported.

## Introduction

Gluten enteropathy (celiac disease) is a chronic disease and presents as diarrhea, weight loss and anemia. It is histopathologically characterized by flatness in the villi, and atrophy and crypt hyperplasia in the small intestine, especially in the duodenum. After prescribing a gluten-free diet, a dramatic improvement is often observed, first in the clinical findings, and then in the histological appearance. Gluten enteropathy may be associated with dermatitis herpetiformis, cystic fibrosis and sarcoidosis [1,2]. Here we present a patient with gluten enteropathy, familial multiple lipomas and seborrheic keratosis who showed clinical improvement on gluten-free diet which corroborated with the histological improvement in duodenal biopsies.

## Case presentation

**Figure 1:**
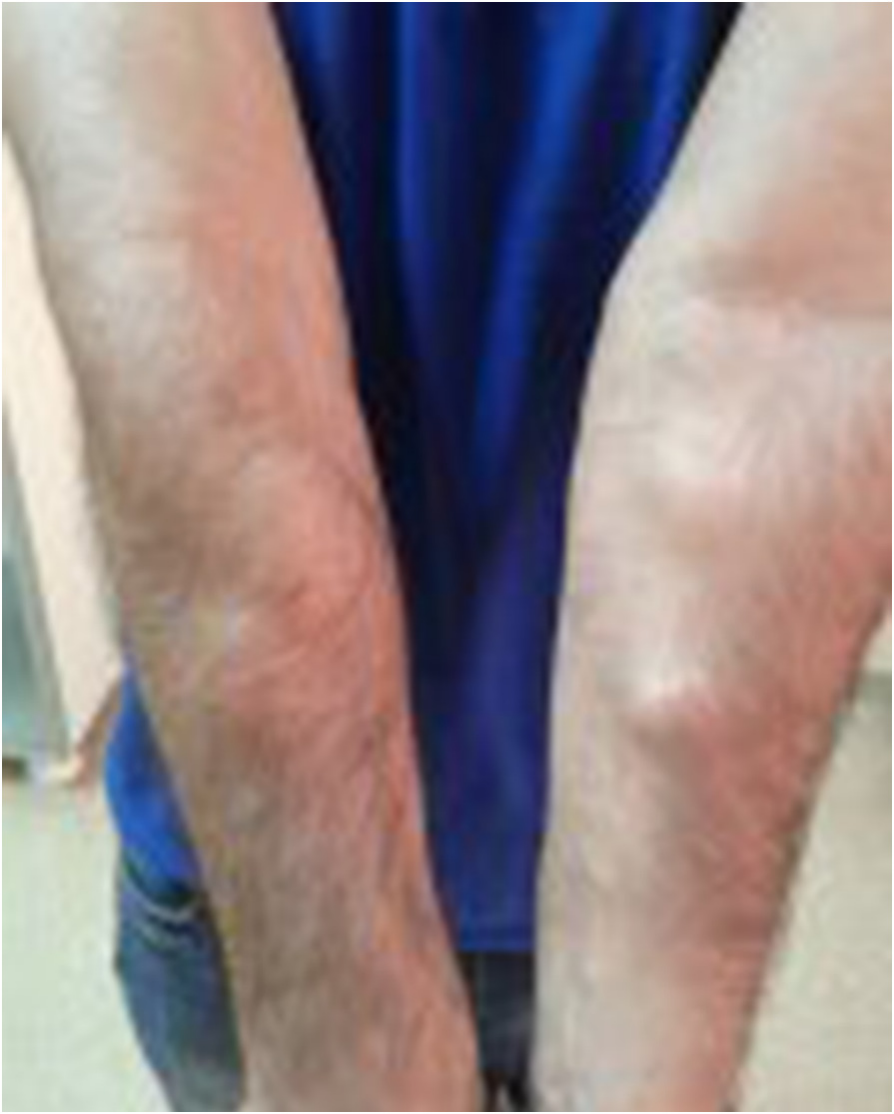
Subcutaneous lipomatous lesions on both upper extremities.

**Figure 2:**
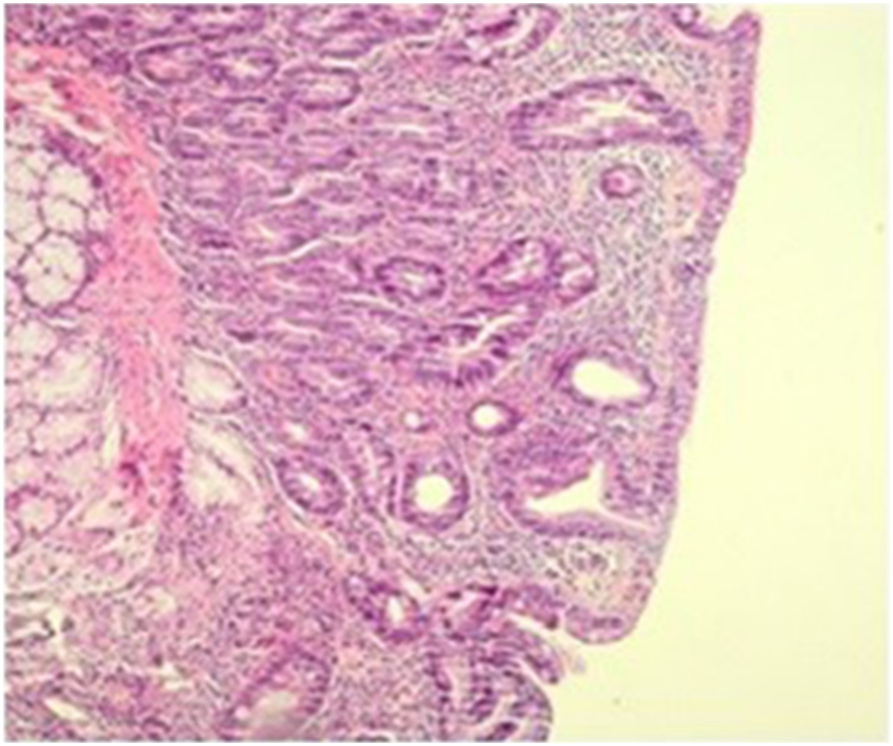
**Villi flattening, cryptic hyperplasia and hypertrophy before treatment.** Hematoxylin and eosin staining, magnification 100×.

**Figure 3:**
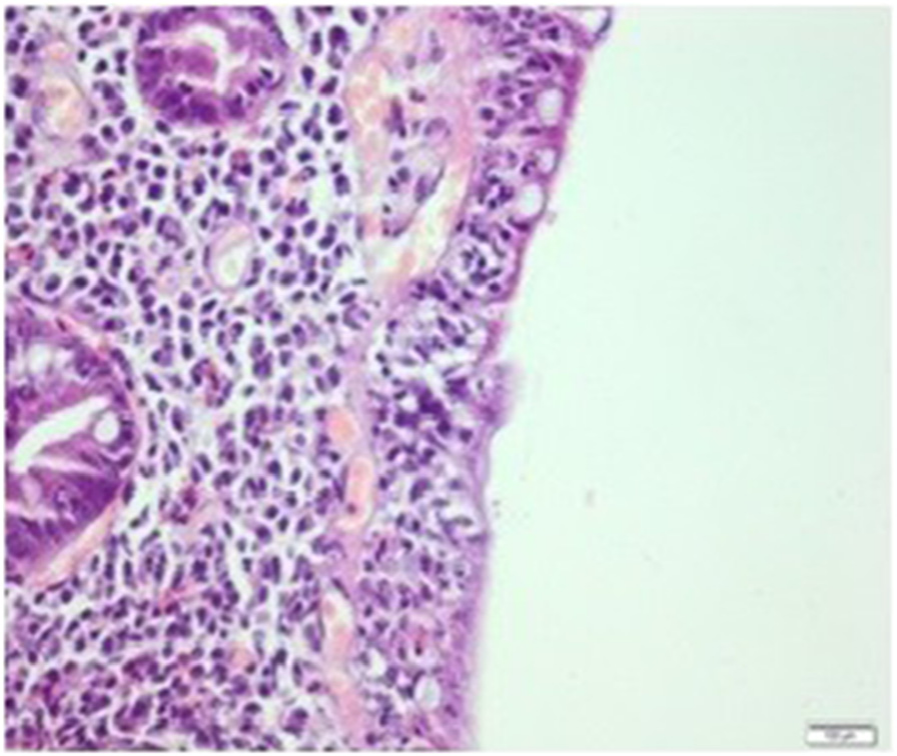
**Intraepithelial lymphocyte increase and severe lymphoplasmacytic inflammatory infiltration in the lamina propria before treatment.** Hematoxylin and eosin staining, magnification 200×.

**Figure 4:**
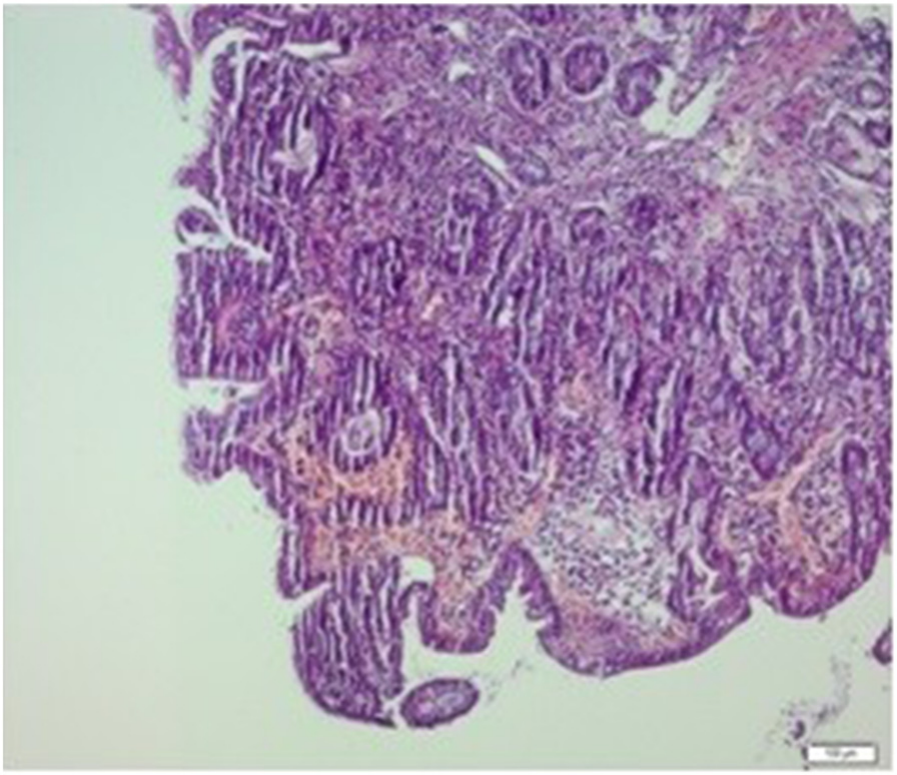
**Slight villi flattening in biopsy after 3 months.** Hematoxylin and eosin staining, magnification 100×.

**Figure 5:**
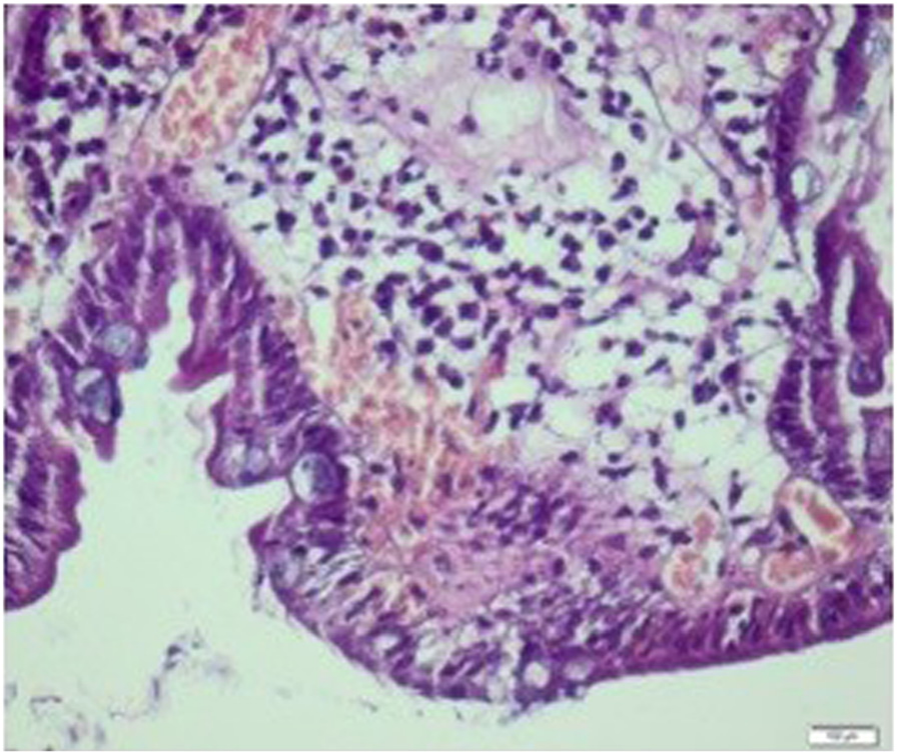
**Mild lymphoplasmacytic inflammation and significant decrease in intraepithelial lymphocytes in biopsy after 3 months.** Hematoxylin and eosin staining, magnification 200×.

A 35-year-old Caucasian man was admitted to the internal medicine clinic of our hospital with diarrhea, weight loss and abdominal pain. He had abdominal pain after food intake and had to go to the toilet after food intake. His stools were greenish and foul smelling and the abdominal pain subsided after defecation. He was admitted to our hospital 1.5 years ago for the same complaints. On physical examination mild tenderness was noted in his abdomen. Multiple painful subcutaneous lipomas of variable size were noted in his arms (Figure 1) and legs. In addition, many papular lesions were found on the skin of his pubic region. His sister, his sister’s children and his uncle’s son had histories of celiac disease. In addition, his mother, brother, sister, his sister’s child and his uncle’s son had a history of multiple lipomas. The results of his blood tests and stool tests were within normal limits. Antigliadin IgA, antigliadin IgG and antiendomysium IgA antibodies (anti-EMA) were positive. A gluten-free diet was suggested for the patient, and his multiple subcutaneous lesions were referred to general surgery.In the first duodenal biopsies, four tissue samples, which were 0.2cm by 0.1cm in diameter and beige in color, had completely flattened villi and an atrophic appearance upon microscopic examination (Figure 2). Intraepithelial lymphocytes were present at a ratio of 45%, and a severe inflammatory lymphoplasmacytic infiltration was present in the lamina propria (Figure 3). After 3 months, slightly flattened villi were observed under the microscope in the four beige tissue samples from the control biopsy, which were 0.2cm by 0.1cm in diameter (Figure 4). In the lamina propria, the lymphoplasmacytic inflammatory infiltration was mild and the presence of intraepithelial lymphocytes was observed at a ratio of approximately 15% (Figure 5).

**Figure 6:**
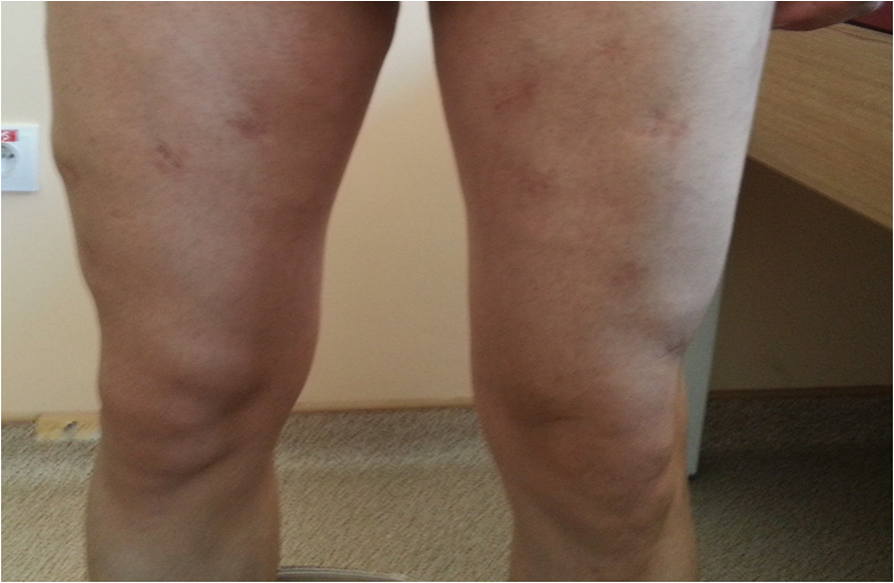
Healed lesions at lower limbs.

**Figure 7:**
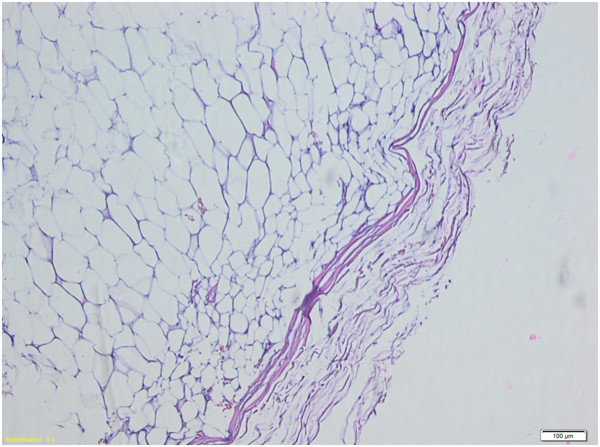
**Histopathological appearance of lipomas.** Hematoxylin and eosin staining, magnification 100×.

**Figure 8:**
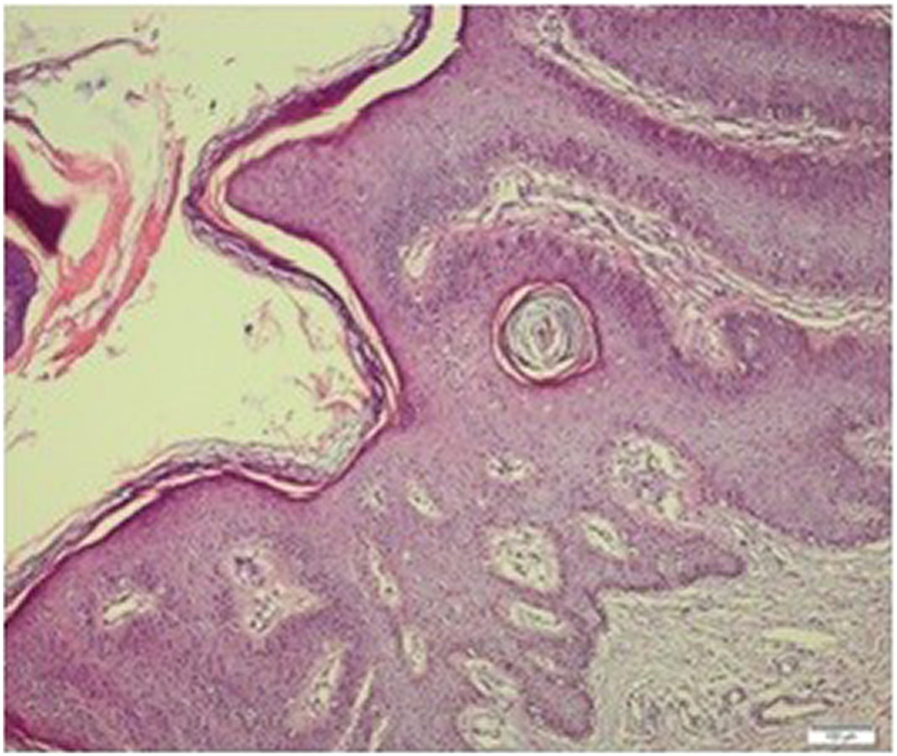
**Microscopic view of lesion in pubic region.** Hematoxylin and eosin staining, magnification 100×.

One month later, the lesions of his upper and lower limbs (Figure 6) were excised by general surgery, with a prediagnosis of lipoma, and sent to the pathology laboratory. In macroscopic appearance, 33 mostly encapsulated solid yellow masses were observed from 0.3cm to 5cm in diameter. On histopathological examination the tumors were encapsulated and contained lipocytes (Figure 7). These lesions, which were taken from his legs and thighs, were reported as multiple lipomas. In addition, skin biopsies were taken from the lesions in his pubic region for histopathological examination during this operation (Figure 8). One skin biopsy that was 0.4cm in diameter was histopathologically reported as seborrheic keratosis.

## Discussion

Celiac disease is a chronic reversible enteropathy caused by gluten sensitivity; it is a polygenic disorder associated with HLA-DQ2/HLADQ8. It presents as chronic diarrhea, abdominal pain, weight loss and anemia. Clinically, a dramatic response to a gluten-free diet is important to confirm the diagnosis. The sensitivity and specificity of classic antigliadin IgA and IgG serological tests are low, and antiendomysium and transglutaminase IgA or IgG antibodies may be used [2]. The histology is characterized by increased intraepithelial lymphocytes in the lamina propria, increased inflammatory infiltration, villous atrophy, crypt hyperplasia and changes in the enterocytes.

In gluten disease, a gluten-free diet may first cause a reduction in the patient’s complaints (diarrhea, abdominal pain, and so on), with the surface epithelial damage being reduced in the following days. Then, chronic inflammation will decrease with increased intraepithelial lymphocytes. After approximately 3 months, the villi will have mostly returned to normal, the crypt hyperplasia will be lost, mitosis will have decreased, and chronic inflammation will be minimal [2]. In our case, the complaints decreased 1 week after starting a gluten-free diet. After 3 months, the histological properties of the intestinal mucosa were normal, with 20% intraepithelial lymphocytes in the control endoscopic biopsy.

Approximately 5 to 8% of all patients with lipomas have multiple tumors that macroscopically and microscopically are indistinguishable from solitary lipomas and there is a defined hereditary basis in approximately one-third of these patients [3]. Although the term “lipomatosis” was used for multiple lipomas, this was suggested because of the excessive overgrowth of the mature adipose tissue [3]. Multiple lipomas that can reach up to a few hundred have been seen, especially in the upper body, shoulders, back and upper arms. Local excision and suction are the treatment options for most lipomas [4]. An inherited property has been identified in one-third of the patients [5,6] and most of the cases were reported as autosomal dominant [7]. In these patients, mutations in the transfer ribonucleic acid genes in the mitochondrial DNA have been identified.

The association of the disease with hypercholesterolemia, diabetes and pregnancy has been reported [8]. In addition, associations with macrocephaly and hemangioma in Bannayan–Zonana syndrome, hemangioma, goiter, skin and mucosal lichenoides, and papular and papillomatous lesions in Cowden syndrome have been seen. *PTEN* gene mutations have been identified in these syndromes [9-11], and in addition to multiple lipomas are the components of Fröhlich and Proteus syndromes [12,13]. In our case, the condition may be from the mitochondrial DNA (of maternal origin) because of the history of multiple lipomas in the maternal relatives. The maternal relatives of the patient also had a history of celiac disease.

Celiac disease has been reported in different skin lesions, including follicular keratosis. On occasion, the histopathological diagnosis of seborrheic keratosis can be confused, especially with condyloma acuminata. It can also be diagnosed with papillomatous hyperkeratosis and koilocytic cell changes that are usually seen on the penis or in the perianal region. However, it has been reported that the human papillomavirus (HPV) screening method may be done with *in situ* hybridization or molecular tests in these condyloma lesions because of the difficulties in the absence of koilocytosis [14]. In one study, HPV DNA (HPV-6) was observed in 72% of cases of vulvar seborrheic keratosis, and this virus was positive in 15% of cases of non-genital seborrheic keratosis. Therefore, most of the vulvar seborrheic keratoses were seen as mature condylomas [15]. Although findings of seborrheic keratosis were observed in the lesions, we detected the presence of koilocytic changes with careful examination using light microscopy in our case.

## Conclusions

Celiac disease is associated with many systemic diseases and some skin lesions; however, its association with familial multiple lipomas has not been documented. We think this was the first case of familial celiac disease with familial multiple lipomas associated with seborrheic keratosis, and we concluded that it was pivotal for the literature.

## Consent

Written informed consent was obtained from the patient for publication of this case report and any accompanying images. A copy of the written consent is available for review by the Editor-in-Chief of this journal.

## Competing interests

The authors declare that they have no competing interests.

## Authors’ contributions

IS, MG, and AI drafted the manuscript. IS, MG, and AI participated in the design of the study. IS, LD, MG, AI, KP, and BG conceived of the study, and participated in its design and coordination and helped to draft the manuscript. All authors read and approved the final manuscript.
